# Supplementary material for: Novel Driver Strength Index highlights important cancer genes in TCGA PanCanAtlas patients
Source: PeerJ. 2022 Aug 11;10:e13860. doi: 10.7717/peerj.13860 (PMC9375969; doi:10.7717/peerj.13860)
Supplement: Supplemental Information 2 [file peerj-10-13860-s002.zip › COHORTS/genes plots/gender/2021_8_16_14_9_distribution_gender_MALE.pdf]

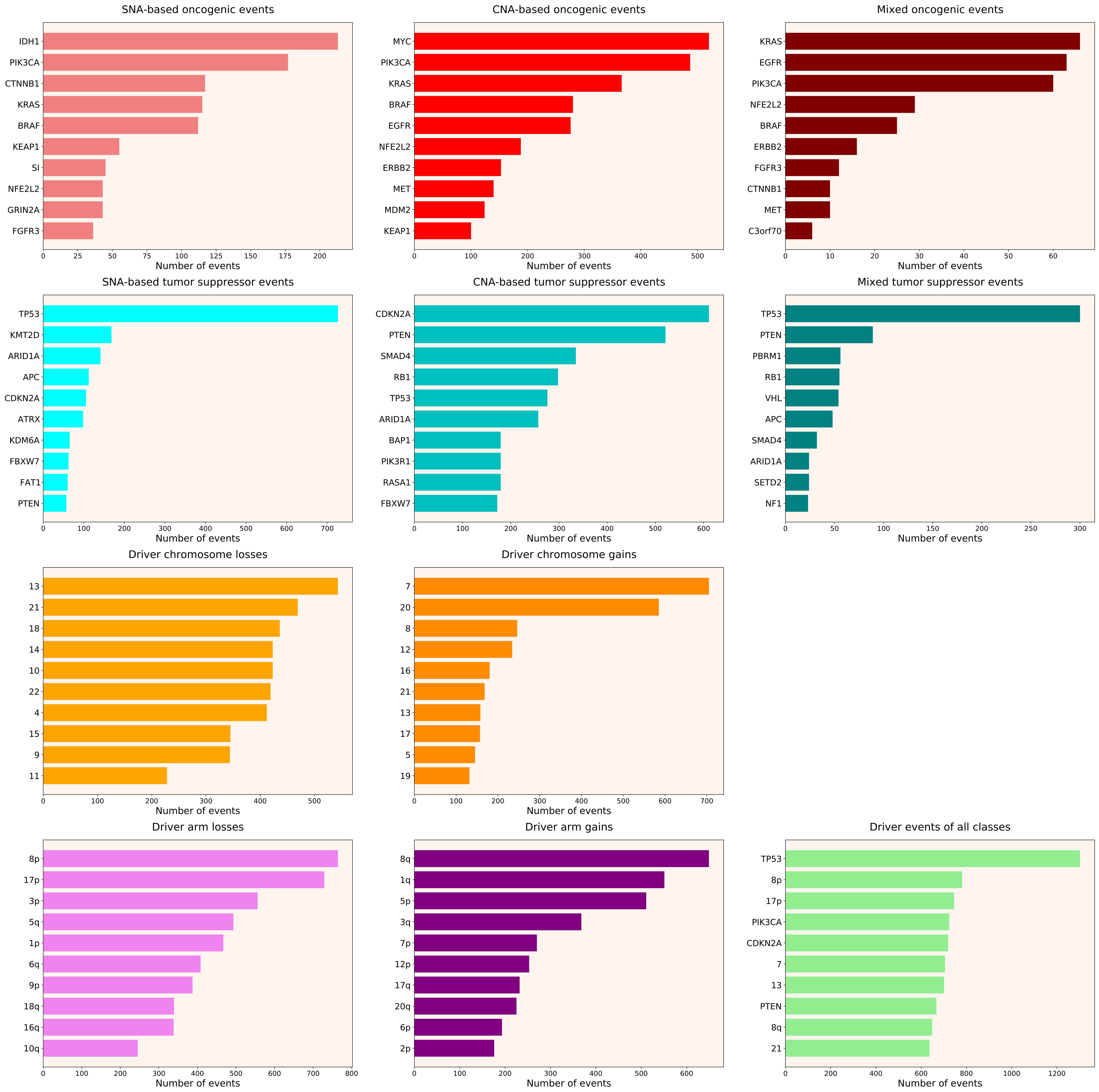

SNA-based oncogenic events
IDH1
PIK3CA
CTNNB1
KRAS
BRAF
KEAP1
SI
NFE2L2
GRIN2A
FGFR3
0 25 50 75 100 125 150 175 200
Number of events
CNA-based oncogenic events
MYC
PIK3CA
KRAS
BRAF
EGFR
NFE2L2
ERBB2
MET
MDM2
KEAP1
0 100 200 300 400 500
Number of events
Mixed oncogenic events
KRAS
EGFR
PIK3CA
NFE2L2
BRAF
ERBB2
FGFR3
CTNNB1
MET
C3orf70
0 10 20 30 40 50 60
Number of events
SNA-based tumor suppressor events
TP53
KMT2D
ARID1A
APC
CDKN2A
ATRX
KDM6A
FBXW7
FAT1
PTEN
0 100 200 300 400 500 600 700
Number of events
CNA-based tumor suppressor events
CDKN2A
PTEN
SMAD4
RB1
TP53
ARID1A
BAP1
PIK3R1
RASA1
FBXW7
0 100 200 300 400 500 600
Number of events
Mixed tumor suppressor events
TP53
PTEN
PBRM1
RB1
VHL
APC
SMAD4
ARID1A
SETD2
NF1
0 50 100 150 200 250 300
Number of events
Driver chromosome losses
13
21
18
14
10
22
4
15
9
11
0 100 200 300 400 500
Number of events
Driver chromosome gains
7
20
8
12
16
21
13
17
5
19
0 100 200 300 400 500 600 700
Number of events
Driver arm losses
8p
17p
3p
5q
1p
6q
9p
18q
16q
10q
0 100 200 300 400 500 600 700 800
Number of events
Driver arm gains
8q
1q
5p
3q
7p
12p
17q
20q
6p
2p
0 100 200 300 400 500 600
Number of events
Driver events of all classes
TP53
8p
17p
PIK3CA
CDKN2A
7
13
PTEN
8q
21
0 200 400 600 800 1000 1200
Number of events
